# Study on Physicochemical Properties of Food Protein

**DOI:** 10.3390/molecules28248145

**Published:** 2023-12-18

**Authors:** Shudong He

**Affiliations:** School of Food Science and Biological Engineering, Hefei University of Technology, Hefei 230009, China; shudong.he@hfut.edu.cn

## 1. Introduction

As the global population continues to grow, the demand for sustainable and nutritious food sources has never been higher. Edible proteins, derived from both plant and animal sources, play a crucial role in providing essential amino acids and contributing to the overall quality of our foods. Thus, the food industry has witnessed a surge of interest in understanding and utilizing the physicochemical properties of proteins derived from various sources, and the study of food protein properties is vital, which in turn, can lead to the development of food products with improved nutritional profiles, enhanced functionalities, and extended shelf lives [1]. Then, the solubility, viscosity, water/oil-holding capacity, gelatinization, and emulsifying and foaming properties of the novel nutritional proteins were widely explored regarding their potential roles in the presentation of texture, flavor, color, and nutrition of foods [2].

Furthermore, recent studies found that the functionality of unfolded or hydrolyzed proteins was different from that of unchanged proteins [3], which revealed the structure–function relationship of proteins. From a structural perspective, studies regarding protein conformation, aggregation, molecular weight, isoelectric point, surface hydrophilicity/hydrophobicity and protein binding characteristics are urgently needed to overcome the knowledge gap between protein exploration and application [4]. Actually, the physical and chemical actions during food processing, such as the mechanical functions, thermodynamic actions, pressure effects, pH, and ionic strength, lead to structural changes in the food proteins as the ingredients further influence the functionality and quality of the food products. In recent research, the effects of novel thermal food processing technologies, such as ultra-high temperature short-time (UHT) heating, extrusion, and microwave heating, as well as non-thermal treatments including high hydrostatic pressure, ultrasound, gamma irradiation, and high voltage electrical discharge, on whey and milk proteins have been preferentially investigated, and the physicochemical and structural properties of related proteins are well known [5]. Moreover, high hydrostatic pressure, ohmic heating, pulsed electric fields, and other emerging processing treatments are called “green technologies”, as they also bring new opportunities to avoid the excessive use of water, gas emissions, and energy [6]. In order to improve or create new protein processing properties, physical, chemical, and enzymatic treatments and combined processing have been widely applied in protein modifications. The physical modifications, including temperature, pressure, shear, pH, ions, and electrostatics, might induce structural perturbation in the thermodynamic state of the protein. The chemical modifications have involved glycosylation, acetylation, succinylation, phosphorylation, and lipophilization, which will change the net charge of proteins via binding with amino acids. Enzymic modification is characterized by enzyme specificity, hydrolysis degree, and protease action site, and the functional properties of protein hydrolysates can be controlled under controlled proteolysis, while the protein hydrolysates can also be used for further food development [7,8,9]. Also, in the past few decades, a number of protein allergens have been identified in foods that induce IgE antibody production and the occurrence of food allergies, and a better understanding of the protein structures and amino acid sequences of the epitopes is crucial for food safety consumption [10].

In this Special Issue, “Study on Physicochemical Properties of Food Protein,” ten outstanding articles were gathered presenting the latest research and innovations in the related field of food science and technology, focusing on the modification, structure, and application of proteins. The ten articles showcase the potential of proteins as functional ingredients in food products, highlighting their impact on flavor, texture, and stability. These studies not only contribute to our fundamental understanding of food proteins but also offer valuable insights for the food industry in terms of food development and process optimization.

## 2. An Overview of Published Articles

Rice protein is a quality, gluten-free, plant-based protein with high biological functionalities and low allergenicity. Yang et al. (contribution 1) examined the factors contributing to the limited solubility of rice protein, which included the abundance of hydrophobic amino acid residues, disulfide bonds, and intramolecular hydrogen bonds, and raised the corresponding modification strategies, such as glycosylation, interaction between polyphenols and heterologous proteins, phosphate modification, protein deamidation, enzymatic hydrolysis with the assistance of heating, ultrasound, microwave, ultra-high pressure, enzymes, and alkaline conditions. Moreover, the application of modified rice proteins in dairy, meat, and baked food products was illustrated due to their good solubility, emulsifying properties, gelling properties, water-holding capacity, and oil-holding capacity.

Low-frequency ultrasound (20–100 kHz, 1–1000 W/cm^2^), a cost-effective non-thermal process, is increasingly employed in the food industry, and the electrical energy is transmuted into vibrational energy, which will generate an acoustic cavitation effect with sudden localized heat and pressure within a liquid setting to promote bond disruption and formation of–diverse covalent and non-covalent linkages, including hydrogen bonds and hydrophobic affinities, with the release of significant energy (10–100 kJ/mol) and physical shearing forces [11]. Liu et al. (contribution 2) explored the potential effects of ultrasonication on the structural integrity and self-assembly behavior of the extracted bone collagen I. The article found that ultrasound (≤200 W, ≤15 min) uniformly disintegrated collagen clusters, significantly escalating collagen self-assembly at a reduced yet consistent fibril diameter and decreasing D periodicity, compared to untreated samples; simultaneously, ultrasound (≤200 W, ≤15 min) diminished viscoelasticity index and gel strength, heightening thermal stability while fostering larger specific surface areas and increased porosity in collagen fibril gels to manufacture novel collagen biomaterials with desirable performances. In contribution 3, ultrasonic (240 W) and transglutaminase (20 U/g egg white protein) treatments were applied to destroy the globular structure of egg white protein, which has an abundance of hydrophobic amino acids and could be a potential emulsifier after modification, and an egg white protein W1/O emulsion was created using 4 wt.% egg white protein gel particles diluted in 40% (*v*/*v*) soya oil. Then, 0.6% (*v*/*v*) of chitosan solutions (W2) was incorporated into this Pickering emulsion to yield an egg white protein–chitosan bilayer emulsion via high-speed homogenization, which would benefit eco-friendly techniques in egg processing, packaging, and distribution.

Recent studies on this topic have researched flavor, color, and the bioactive properties’ promoting effects of Maillard reaction products (MRPs) derived from protein hydrolysates, comprising a blend of peptides and amino acids [12]. The impact of ultrasound on the characteristics of MRPs derived from hydrolyzed soybean meal was studied in contribution 4. Prior to the Maillard reaction, the Maillard reaction system, with the addition of cysteine (2.0%), xylose (3.0%), VB1 (0.05%), and enzymatically hydrolyzed lard (1.0%) into the soybean meal enzymatic hydrolysis, was sonicated at 300 W (5 s on; 5 s off) for 30 min. The article revealed that ultrasonic processing escalated unsaturated fatty acid production within the aqueous phase during the lard hydrolysis and had boosting effects on Maillard reaction intermediates and melanoid accumulation in the product, and then both the quantity and flavor-enhancing components of volatiles were enhanced after the ultrasound while the excellent antioxidant features were demonstrated.

In particular, the application of limited hydrolysis and ultrafiltration strategies has exhibited promising results in enhancing the physico-functional attributes of food proteins. In contribution 5, donkey-hide gelatin was treated by the Alcalase 2.4L, and the structural characteristics and antioxidant activities of five molecular weight (MW) hydrolysates, including >30, 10–30, 3–10, 1–3, and <1 kDa fragments, were evaluated. The peptide of MW 1–3 kDa presented the strongest antioxidant activity, which was attributed to the amino acid composition. Furthermore, the amino residues of Arg415, Gly462, Phe478, and Tyr572 were confirmed as the key sites to bind with the Keap1 protein via molecular docking, indicating good potential for antioxidant capacity.

The food processing procedure combining multiple mechanical forces has attracted a lot of attention as it is highly efficient in protein modification. Xu et al. (contribution 6) utilized ultra-high-pressure jet processing on skimmed bovine milk. This approach altered the secondary and tertiary casein structures, contributing to a decrease in the mean particle size and zeta potential of casein micelles. Consequently, flatter, looser, and porous casein micellar morphologies improved curd production efficiency and the texture of fermented milk. In contribution 7, steam flash explosion (SFE) was applied to modify high-temperature denatured defatted rice bran protein isolate (RBPI), and the surface hydrophobicity and thermal stability decreased with an increase in the intrinsic viscosity resulting from the protein unfolding. Then, high-temperature rice bran proteins could be utilized in the food industry with their enhanced solubility. 

In contribution 8, the hot-pressed peanut meal was used to produce the plant protein-based adhesive via sodium dodecyl sulfate (SDS) denaturation and polyamide epichlorohydrin (PAE) crosslinking. Papain hydrolysis contributed towards enhancing wet shear strength, whereas SDS and urea disrupted hydrogen bonds for better water resistance. Significantly, PAE amplified both the wet shear strength and water resistance of the adhesive with an increase in viscosity.

In the study presented in contribution 9, esterification was applied to soy proteins, leading to a notable increase in the isoelectric point (pI) of the esterified proteins. This increase was attributed to the formation of esters between alcohol and carboxyl groups, which subsequently reduced the content of negatively charged groups on the protein surface. Consequently, the esterified soy protein nano-emulsions demonstrated remarkable storage stability, freeze–thaw stability, and thermal stability. Furthermore, the esterification modification imparted antibacterial properties. These findings highlight the potential of esterified soy proteins as a promising material in various applications, particularly in the food and pharmaceutical industries.

As one of the nine major food allergens identified by the Food and Drug Administration in the USA, it becomes imperative to elucidate the primary allergenic epitopes of cashew nut proteins. Zhang et al. (contribution 10) utilized DNAstar and PyMoL tools to ascertain potential epitopes for Ana o 2 and Ana o 3 proteins. They then applied the phage display methodology to validate the prospective synthetic epitope peptides. It is noteworthy that Ana o 3 might carry a greater risk of provoking an allergic response compared to Ana o 2, which is primarily attributed to the larger number of identifiable epitopes.

## 3. Conclusions

The ten research contributions presented in this issue collectively demonstrate the significance and versatility of studying the physicochemical properties of food proteins under various modifications. These studies have explored various aspects, including protein solubility, structural integrity, emulsifying properties, and allergenic epitopes, as well as the application of different processing techniques such as ultrasonication, high-pressure processing, and esterification. Advances in “eco-friendly” technologies provide promising opportunities to diminish water consumption, gas emission rates, and energy utilization within the food industry.

The findings from these studies have not only contributed to a deeper understanding of the role of proteins in food products, but they have also provided valuable insights for the food industry in terms of product development, process optimization, and the potential application of novel protein-based materials. For instance, the use of modified rice proteins in dairy, meat, and baked goods; the prospective use of low-frequency ultrasound in protein manipulation; taste enhancement provided by amino acid compounds resulting from the Maillard reaction; the development of plant protein-based adhesives; the potential application of esterified soy proteins; and limited enzymolysis donkey-hide gelatin in the food and pharmaceutical industries. Furthermore, pioneering processing methodologies like ultra-high-pressure jet processing and steam flash explosions exhibit their effectiveness in modifying protein architectures for better functionality. Of particular interest, the analysis of soybean protein esterification uncovers its potential application in nano-emulsion formations with improved durability and antibacterial properties. The recognition of allergenic epitopes in cashew nut proteins accentuates the necessity of comprehending allergenicity to safeguard consumer well-being.

In conclusion, the research presented in this issue showcases the potential of food proteins and their modifications in various applications, emphasizing the need for continued research and innovation in this field. By further exploring the physicochemical properties of food proteins and developing new processing techniques, the food industry can continue to create high-quality, nutritious, and safe products that cater to the diverse needs of consumers.
